# The aftermath of the Merck's HIV vaccine trial

**DOI:** 10.1186/1742-4690-5-56

**Published:** 2008-07-02

**Authors:** Enrico Iaccino, Marco Schiavone, Giuseppe Fiume, Ileana Quinto, Giuseppe Scala

**Affiliations:** 1 Department of Experimental and Clinical Medicine, University of Catanzaro "Magna Graecia", 88100, Catanzaro, Italy; 2 Department of Biochemistry and Medical Biotechnology, University of Naples "Federico II", 80131, Naples, Italy

## Abstract

The recently released results of the Merck's Phase IIb "test-of concept" vaccine trials have shown no protection from HIV-1 infection in the vaccinated group compared with a control group vaccinated with placebo. The study was designed to test the Merck's MRKAd5 trivalent candidate vaccine. The vaccine formulation was expected to stimulate a HIV-specific T cell immune response and to either prevent infection, or to reduce the levels of the viral load in vaccinated subjects. Upon the first evaluation of the interim data, the independent Data and Safety Monitoring Board (DSMB) underscored no protection from HIV-1 infection in the vaccine-inoculated volunteers compared with the control group; accordingly, the vaccine trial was stopped. This disappointing outcome warrants a critical analysis of the current vaccine studies and calls for a renewed effort toward a rational design of novel immunogens to be tested in large primate trials.

## Background

The release of the results from the phase III efficacy trial sponsored by Merck, HIV Vaccine Trials Network (HVTN) and the NIAID-NIH has shattered the community of scientists engaged in the field of HIV vaccine. The STEP vaccine trial (also referred to as HVTN 502 or Merck V520-023) relied on recombinant adenovirus serotype 5 (rAD5) as a vaccine vector to induce a strong anti-HIV T-cell immunity. The trial included three rAD5 vectors expressing *gag*, *pol *or *nef *coding sequences and was expected to either prevent vaccinees from HIV infection, or to reduce significantly the plasma viral loads at post-infection set point. The STEP study enrolled 3,000 volunteers at sites in Australia, Brazil, Canada, the Dominican Republic, Haiti, Jamaica, Peru, Puerto Rico and the United States. The first set of results, released in September 2007, showed no protection from infection in rAD5-gag, rAD5pol and rAD5nef vaccinated individuals; surprisingly, the vaccinated cohort showed an increased number of new infections compared with the control group [1]. In fact, an independent Data and Safety Monitoring Board (DSMB) found 24 cases of HIV infection among the 741 volunteers who received at least one dose of the investigational vaccine compared with 21 cases of HIV infection among the 762 volunteers who were vaccinated with placebo. In volunteers who received at least two vaccinations, the DSMB found 19 cases of HIV infection among the 672 volunteers who received the investigational vaccine and 11 instances of HIV infection among the 691 subjects who received the placebo. Surprisingly, the STEP results underscored that people who got the vaccine were more likely to get infected with HIV. Moreover, the increased susceptibility to HIV was preeminent in subjects with pre-existing immunity (antibodies) to adenovirus type 5 (Ad5) (Table 1). As a consequence, enrollments and vaccinations were discontinued. It is noteworthy that the STEP trial was stopped before completing the immunizations in most of the volunteers and that the reported infections occurred in subjects with incomplete vaccine regimen, thus precluding a fair evaluation of the vaccine protection. In addition, the control groups were not inoculated with empty vector and do not act as true controls to evaluate the potential enhancement of infection. Nevertheless, the available results do suggest that multiple immunizations with a single immunogenic vector may stimulate a harmful anamnestic response.

**Table 1:** Rates of HIV-1 infections in the vaccinees and in placebo inoculated volunteers enrolled in the STEP vaccine trial.

	No immunity to Ad5 (< 18 units)	Medium immunity to Ad5 (18–200 units)	High immunity to Ad5 (201–1000 units)	Very high immunity to Ad5 (> 1000 units)
Vaccine^a^	20 infected out of 382 total	8 infected out of 140 total	14 infected out of 229 total	7 infected out of 163 total
Placebo^b^	20 infected out of 394 total	4 infected out of 142 total	7 infected out of 229 total	2 infected out of 157 total

a. Subjects were inoculated with three rAD5 vectors expressing *gag*, *pol *or *nef *coding sequences.

b. Control subjects were inoculated with an inactive substance (saline). Immunity to Ad5 vector was assessed by ELISA titers of the antibody response to the rAd5 vector.

## Discussion

**Figure 1:**
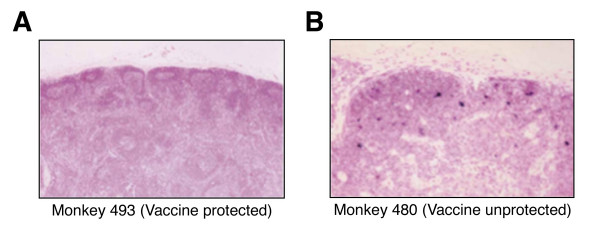
**Lymph node histomorphology and *in situ *hybridization in vaccinated monkeys challenged with SHIV-89.6PD viral RNA**. **A**, Representative lymph node of monkey 493 (vaccine protected) [13]. Inguinal lymph nodes were removed at day 70 after challenge, stained with hematoxylin-eosin and subjected to *in situ *hybridization. The picture shows the conserved lymph-node architecture and the presence of secondary follicles with expanded germinal centers and well-represented paracortical T cell regions. No SHIV viral RNA is detected. **B**, Representative lymph node of monkey 480 (unprotected). The picture shows the follicular depletion, paracortical atrophy and abundant presence of SHIV viral copies. Reprinted by permission from Macmillan Publishers Ltd: Nature Medicine, Chen, X. et al., 2001, copyright 2001, Nature Publishing Group [13].

**Figure 2:**
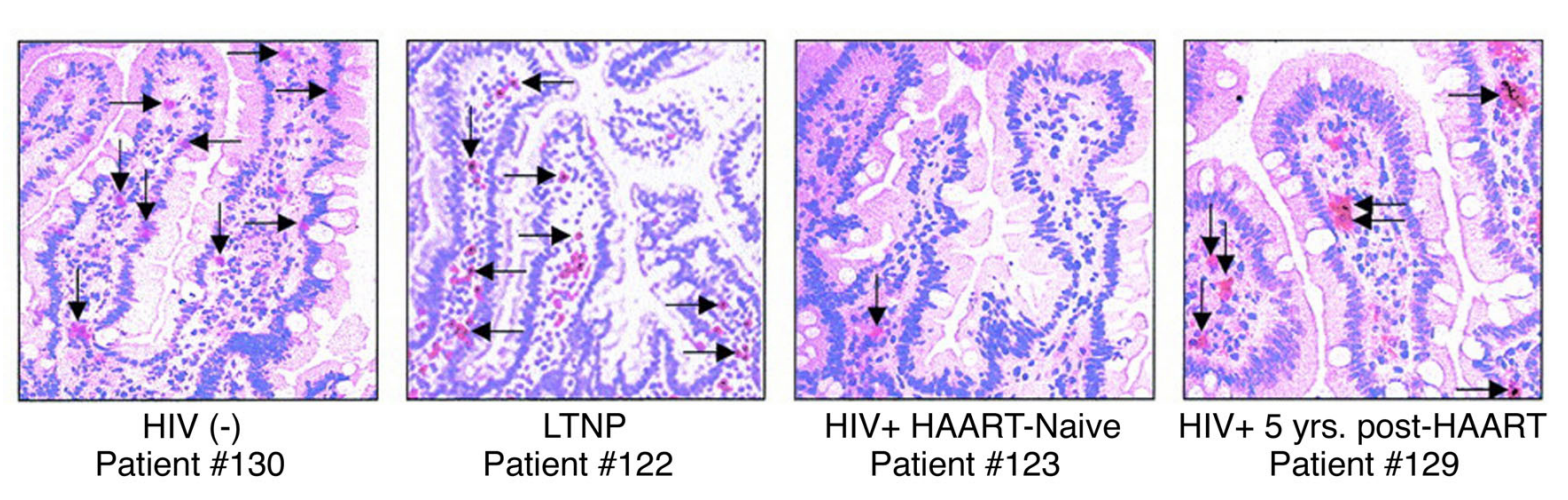
**Immunohistochemical analysis of CD4^+ ^T-cell depletion in gut-associated lymphoid tissue of HIV-1-infected individuals**. Immunohistochemical detection of CD4^+ ^cells of an HIV-negative healthy control, an LTNP, a HAART-naïve chronically HIV-1-infected patient, and an HIV-1-infected patient who had received HAART for 5 years. Reprinted by permission from the American Society for Microbiology: Guadalupe, M et al., 2003, *Journal of Virology *[14].

These results represent a grim setback for the HIV vaccine field; however, they came with no surprise within the vaccine community. Indeed, such an outcome was predicted given the nature of the HIV-1 infection and by previous preclinical trials in monkeys. HIV-1 infection is characterized by an acute phase of infection where the virus integrates into the host CD4-positive cell genome and establishes an early viral reservoir characterized by a HIV latency in memory CD4+ T cells [2,3]. However, upon a stochastic activation of CD4 T cells by multiple stimuli, the HIV latency is overcome by the Tat-mediated transactivation of the LTR, which promotes the virus gene transcription and production of infection-competent viral particles that spread to uninfected T cells thereby reconstituting the viral reservoir [3-6]. Reactivation of viral infection occurs preferentially in activated memory T cells [7-9], and results in a limited T-cell repertoire and in a reduced immune competence [10]. In this setting, the immune system mounts a robust anti-HIV T cells immune response that decreases significantly the acute plasma viremia to a lower set point; however, shortly after the primary infection, the T cell-mediated immune pressure generates escape mutants that mediate new rounds of infection that result in increased virus spreading and further deletion of CD4^+ ^susceptible cells [11-14] (shown in Figure 1, 2).

Antibody-mediated immune response is elicited after few weeks from primary infection; however, the anti-HIV antibodies show a poly-specificity for non-neutralizing, linear epitopes [15]. Antibodies specific for conserved, conformational epitopes are elicited at later stage and may play a favorable role by decreasing the plasma viremia [16-18]. In addition, anti-HIV envelope antibodies also contribute to the containment of infected T cells by triggering antibody-mediated cell cytotoxicity (ADCC; [19]). As in the case of virus escape mutants promoted by the T-cell immune pressure, escape mutants are generated in presence of epitope specific antibodies [20].

In this setting, most vaccine trials in humans have been initiated under the assumption that the immune response to HIV-1 differs from the one elicited in non-human primates upon infection with SIV or SHIV strains; by inference, only vaccine trials in humans would validate vaccine candidates. Consistently, a large number of monkey vaccine studies have been overlooked despite the evidence that the monkeys' models of SIV/SHIV infection mimic well the natural HIV-1 infection in human subjects. Indeed, monkeys infected with SIV or SHIVs show levels of viremia comparable with the ones experienced by HIV-infected subjects in the course of the primary infection and at the subsequent set points [21,22]; CD4 activated T cells are the main susceptible cell type and the involved anatomical districts, mucosal sites and lymphoid tissues, are similar [2]; the functional characteristics of the monkeys' immune response do not differ significantly from the one elicited in humans both at the time of primary infection and at the later stage of the immune deficiency [13,23]; the T cell-mediated and the antibody immune response are comparable with the ones elicited in humans infected with HIV-1 strains and give rise to escape viral mutants, as reported in HIV-1 infected subjects [12,23,24]. Relevant to the vaccine field, several monkey studies have documented that passive transfer of neutralizing MAbs induced protection from systemic and mucosal challenge [25-27]. This evidence indicates a narrow path to a successful HIV vaccine based on the induction of neutralizing antibodies focused on conserved, constrained envelope domains.

The results coming from previous studies in monkeys, including the ones promoted by Merck in collaboration with academic laboratories, may have been largely ignored. For example consider the world's first 2 phase 3 of HIV-1 vaccine efficacy trials that involved two large cohorts of HIV-1-uninfected volunteers and were completed in 2003 [28]. The first trial (VAX004) included 5403 individuals from North America and The Netherlands; the cohort included both men who have sex with men and 308 women at high risk for heterosexual transmission. The second trial (VAX003) was conducted in 2527 infection drug users subjects in Thailand [29,30]. Both the studies relied on recombinant gp120 envelope protein as the vaccine immunogen. Results from both the studies indicated that despite a robust antibody response elicited in vaccinees, no significant protection from HIV-1 infection was detected in the placebo and in the vaccine inoculated groups [28]. These negative results were anticipated by monkeys studies designed to test a variety of envelope proteins as anti-HIV immunogens [31]. Among these pre-clinical studies, the 2001 work of Cho et al. [32] analyzed the efficacy of mono- or polyvalent envelope proteins from distinct HIV clade B strains. This study reported that despite the induction of a strong anti-envelope antibody in vaccinated monkeys, no significant protection from the SHIV challenge was detected [32].

In the case of the Merck's STEP HIV vaccine trial, the rationale was based on a preclinical vaccine study in rhesus macaques monkeys (*Macaca mulatta*) primed with a plasmid expression vector followed by a boost with Ad5, a replication-incompetent adenoviral vaccine vector, which both delivered the SIVmac239 *gag *gene; the monkeys were then challenged with SHIV-89.6P, a pathogenic strain [33]. The reported results showed high levels of CD8+ T-cell response by tetramer staining at around week 35 post-challenge and a preserved CD4 T-cells up to day 180. Peak viremia was in the range of 10^6^–10^7 ^at about week 3, one log lower than the naïve animals; animals were followed up to 120 days [33]. In a distinct study, Barouche et al. reported the case of a monkey immunized with a plasmid expressing SIV *gag *plus IL2-Ig protein and challenged with pathogenic SHIV89.6P [34]; this animal, which showed low levels of plasma viremia at week 10–20, developed an escape mutant of the Gag p11C immunodominant epitope and showed a rapid disease progression and death from AIDS-related syndrome [11]. These reports are fully consistent with the dynamics of immune escape of HIV in infected subjects [4,17] and highlight the need for a vaccine capable of inducing a drastic decrease of the viremia at the primary infection to prevent the development of pathogenic escape mutants and the impairment of the T-cell repertoire.

These representative studies raise the question of the quality and the extent of the induced immune response. Indeed, both in monkeys and in human vaccine trials, positive results were expected from the presence of either a strong anti-envelope antibody response [28], or a robust T-cell mediated immunity [1]. Clearly, the main correlates of protection, anti-envelope antibodies and T-cell induced activation, need to be re-evaluated. In the case of the antibody response, previous studies have assessed the specificities and concentrations of neutralizing Abs required to achieve a complete protection from systemic or mucosal infection [25,26]; however, the major challenge in the field is to devise novel immunogens to induce antibodies that match the epitope specificities and affinity of the few available neutralizing antibodies [35]. In the HIV vaccine field, the T cell-mediated immune response is assessed by ELISPOT and polychromatic FACS analysis to detect cytokine-expressing cells [36] without a functional assessment of T-cell killing activity toward HIV-infected cells. Thus, the functional parameters of successful T-cell activities are still missing and may hamper the predictive value of the ongoing phase 1–2 immunogenicity studies [37-39].

## Conclusion

In the context of the above-mentioned studies, clinical trials have flourished upon societal and economic pressure from geographic regions heavily affected by AIDS, and fueled by a large financial support from public and private institutions [40,41]. However, vaccine trials with the available immunogens in different vector systems may, at best, only moderately decrease the levels of plasma viremia and are not expected to improve the protection from infection. Likewise, any trial with a suboptimal immunogen in a high HIV incidence areas may create a significant number of vaccine breakthroughs and would raise ethical and financial issues concerning the treatment of the infected volunteers [42]. In this context, we should learn from the STEP trial that preclinical studies in monkeys are feasible and may anticipate future results in human subjects. Preclinical studies in non-human primates should be expanded in terms of number of animals, study designs to assess potential negative effects and length of the post-challenge follow-up. Several lessons are coming form the Merck vaccine study:

a. A vaccine aimed at inducing T-cell immunity will not prevent primary infection and the establishment of the viral reservoir;

b. Studies aimed at inducing neutralizing antibodies should be pursued with even greater efforts toward a rational development of structurally defined conformational epitopes, and tested in suitable monkey models;

c. The host immune system will react to both the vector and the immunogen in an unpredictable way, by activating a complex immune response that includes the innate and the epitope-specific immunity;

d. Studies aimed at modeling human behavior in the context of HIV exposure during vaccination should be developed and used to test for potential vaccine-induced enhancement of infection.

The STEP vaccine has not provided a successful road to an effective HIV vaccine; however, it has shown to us that we still know too little and should learn more.

## Competing interests

The authors declare that they have no competing interests.

## Authors' contributions

EI, MS and GF selected the relevant literature. IQ and GS wrote the commentary. All the authors read and approved the final manuscript.
